# Prevalence of obesity among students aged 6 to 12 in China: a meta-analysis

**DOI:** 10.3402/fnr.v59.25747

**Published:** 2015-12-17

**Authors:** Yue Tian, Haixia Wu

**Affiliations:** Department of Pediatrics, Yantaishan Hospital (affiliated with Taishan Medical College), Yantai, China

**Keywords:** primary students, obesity, prevalence, meta-analysis

## Abstract

**Background:**

In recent years, obesity has become a major public health problem worldwide. It has been estimated that 8% of children are obese. This study evaluates the overall prevalence of obesity in primary students in recent years.

**Methods:**

Publications from 2011 and 2015 on the obesity prevalence among primary school students aged 6 to 12 in China were retrieved from the databases PubMed and Wanfang Data. Obesity was defined using the International Obesity Task Force standard body mass index cutoff points established for children. MetaAnalyst 3.13 software was used to calculate the total prevalence of obesity among primary school students in recent years.

**Results:**

After evaluation of the quality of the articles, 14 papers were finally included in our study. The pooled prevalence of obesity in students aged 6 to 12 is 10.2% (95% CI: 7.1–14.6%).

**Conclusion:**

Our results indicate that obesity is prevalent in students aged 6 to 12 in China. We believe that appropriate measures should be taken to control this situation.

Globally, there is a rising prevalence of overweight and obesity in both developing and developed countries (1). Along with developing countries over the past 30 years, recent data suggest that the prevalence of childhood obesity in China is plateauing and may be increasing (2).

Some studies observed that obesity is associated with increased blood pressure (3, 4), breast cancer (5), asthma (6, 7), diabetes mellitus (8, 9), hypertension (10), coronary artery disease (11), and dental caries (12–14). A previous study showed that the prevalence of obesity in Chinese children and adolescents was still considered to be relatively low (15). Obesity has a greater prevalence among white children than among the Han Chinese, exacerbating existing disparities (4, 5).

In the context of these reports, we updated an earlier meta-analysis of obesity among Chinese schoolchildren aged 6 to 12 (16, 17). That earlier analysis found the prevalence of obesity in primary school students to be 10.0% and 10.4% (16, 17). Our objective was to examine obesity trends among children in recent years.

However, there have been few studies documented in the literature in this part of China that assess the prevalence of overall obesity. Thus, the purpose of this study was to assess the prevalence of obesity in the past 5 years.

## Materials and methods

### Literature retrieval

Related publications on obesity published from 2011 and 2015 were selected from the PubMed and Wanfang Data databases with the keywords *obesity*, *students*, *primary*, and *China* in Chinese for the Chinese database and in English for PubMed. Eligible full texts were retrieved manually from the previous data.

### Criteria

The literature met the following criteria: 1) papers included on the obesity among students aged 6 to 12 in China were published between 2011 and June 2015; 2) articles focused on discussion of the prevalence of obesity in China among primary students; and 3) overweight and obesity were defined using the International Obesity Task Force standard (IOTF) body mass index cutoff points established for children (18). Exclusion criteria included 1) the indicators described in articles with less association or data being incomplete; 2) duplicate articles.

### Literature screening and quality assessment in process

Each study was assessed by two investigators independently. Blinding was used to ensure quality. The related literature was retrieved on basis of the keywords described previously and initially selected by appraising the title and scanning the abstracts. Data extraction was performed for papers verified to be eligible. Evaluation of the article quality was performed as a meta-analysis of observational studies in epidemiology proposed by Stroup et al. (19).

### Statistical analysis

MetaAnalyst 3.13 software (20) was used for performing meta-analysis. As a heterogeneity test, the random effects model was applied to evaluate the overall prevalence of obesity in school-aged children.

## Results

### Basic information and quality assessment of the articles

**Fig. 1:**
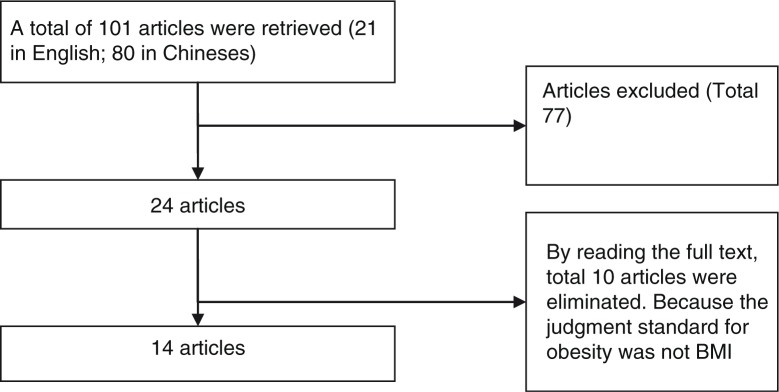
Flowchart illustrating the literature screening.

A total of 131 articles were retrieved from the online Chinese periodical full-text databases VIP, Wanfang Data, and CNKI, as well as PubMed. Quality assessment was made by meta-analysis of observational studies in epidemiology (19). Figure 1 shows the process of literature screening, and the basic information on the final articles is shown in Table 1.

**Table 1:** Characteristics of the studies included

		Geographical distribution	Prevalence of obesity (%)			Age range
		
First author	Year		Total	Boys	Girls	
Zhuang (21)	2011	Chaozhou	10.26	13.58	6.59	7–12
Jiang (22)	2011	Ji'an	11.20	14.50	8.00	7–12
Fu (23)	2011	Shanghai	22.64	–	–	7–12
Huang (24)	2011	Beijing	21.00	28.00	14.40	7–11
Lai (25)	2011	Shenzhen	8.98	10.65	7.19	7–12
Liu (26)	2012	Yongzhou	13.7	21.2	3.9	6–12
Zhu (27)	2013	Zhoushan	12.86	15.93	9.51	7–12
Jia (28)	2013	Wenzhou	6.4	7.9	4.6	6–13
Zhou (29)	2013	Dongguan	7.32	5.55	1.77	7–14
Gao (30)	2013	Suzhou	11.23	14	7.9	7–12
Yao (31)	2014	Tongling	3.66	5.2	1.8	5–14
Hua-Yun (32)	2015	Yunnan	5.53	6.17	4.82	7–12
Bin (33)	2015	Shanghai	22.1	24.8	14.6	8–11
Qiu-Lan (34)	2015	Guangxi	5.38	6.5	4.16	6–12

### Meta-analysis of the prevalence of obesity among primary school students in China

**Fig. 2:**
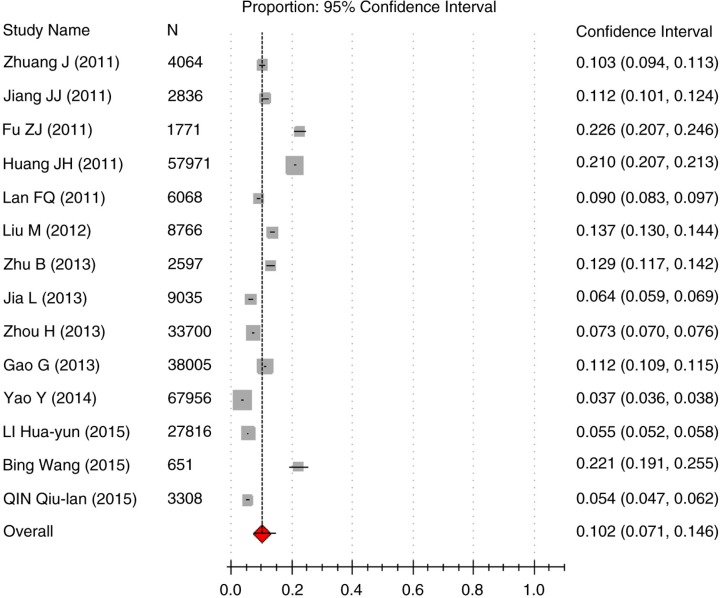
Forest plot of overall prevalence of obesity in the meta-analysis.

A heterogeneity test was carried out on the obesity detection rate, with a result of *I* ^2^=0.500, suggesting that the research results in the 14 papers were heterogeneous. The random effects model was used for meta-analysis. As shown by the forest plot in Fig. 2, the results suggested that the pooled prevalence of obesity in primary school students is 10.2% (95% CI: 7.1–14.6%).

### Publication bias

**Fig. 3:**
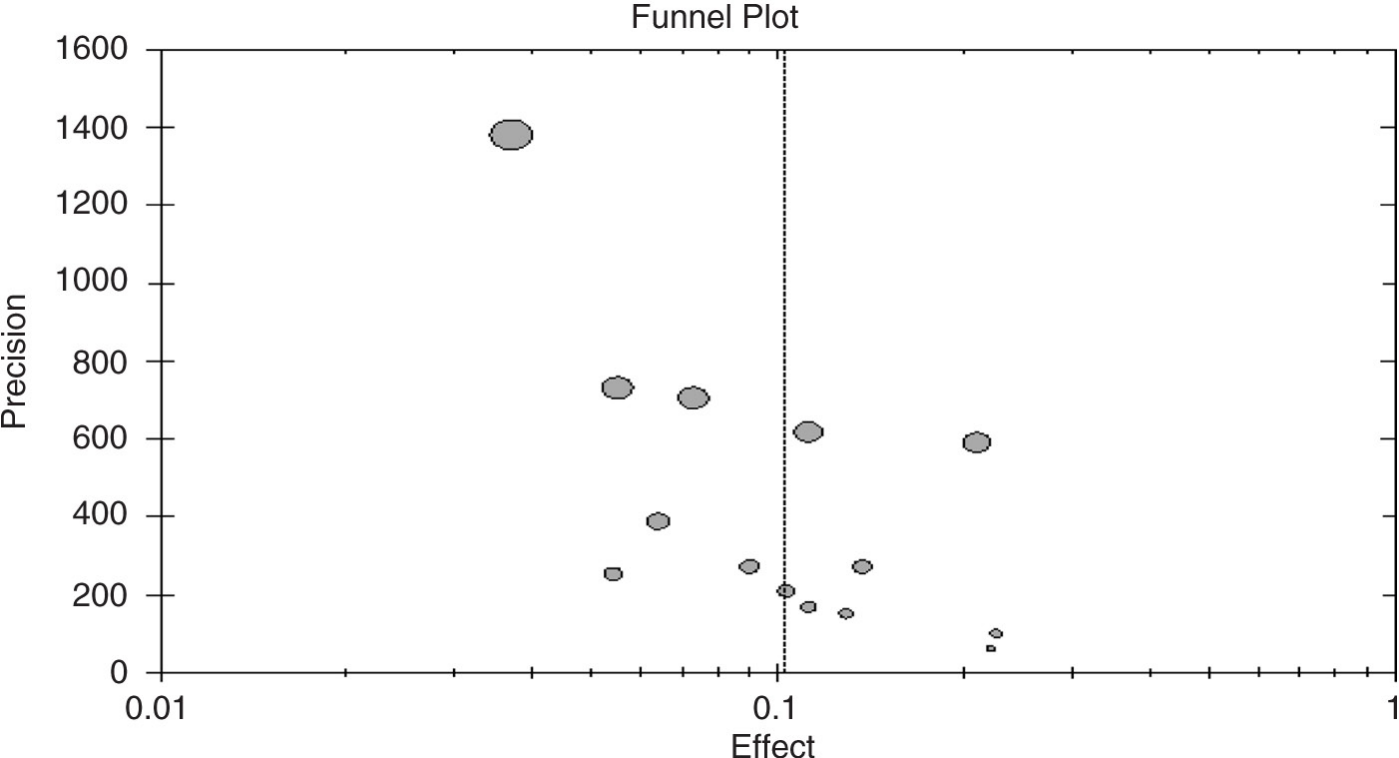
Funnel plot.

Publication bias is a tendency on the part of investigators to submit, or reviewers and editors to accept, manuscripts containing results that appear statistically significant. Although a potential threat in meta-analysis, it may be verified with funnel plots, which were applied to modify the possible bias in our literature selection. Verification by funnel plot (Fig. 3) showed that the literature included was more or less symmetrical.

## Discussion

Our results indicated that the obesity prevalence status in China was still troublesome: the pooled prevalence of obesity in primary school students is 10.2% (95% CI: 7.1–14.6%). This result is consistent with our previous study (16, 17) and lower than the 20.3% obesity prevalence in the United States (35, 36). In Greece, the prevalence of obesity is also high but shows a decreasing trend (37). The prevalence of obesity is different in different areas of China. The overall prevalence rates of overweight and obesity were higher in northern areas than in other areas (38). Detailed reasons for this trend should be investigated in future studies.

Although the figure from our meta-analysis (mean 10.2%) is not as high as reports from Western nations, the prevalence in mainland China is still troublesome, because the situation will worsen if we fail to take timely, effective preventive and control measures. We must be highly aware of the seriousness of the situation, and our educational departments and health authorities should jointly take effective and practical measures, such as health education and regular physical examination. Moreover, parents should be educated about the importance of healthy eating and students encouraged to get more exercise and shape good living habits.

### Limitations

This study provides the status of childhood obesity from 2011 to 2015 in China. Our meta-analysis had some limitations. The data included are from published papers; some publication bias may exist. For example, genetic susceptibility may have an effect on childhood obesity in China. Therefore, obtaining more reliable obesity prevalence data on elementary school students in China requires further investigation. Our conclusions were limited by poor reporting in the studies included. Our review was also limited by significant heterogeneity, which we addressed by random effects meta-analysis and predefined subgroup analyses. This heterogeneity appears to be mainly due to differences in criteria, education level, economic status, and ethics of subjects.

### Strengths of the study

Several important outcomes were reported in this study. The prevalence of obesity in primary school students is moderately high. Although it is lower than in most Western countries, no decreasing trend for obesity prevalence was noted in China.

## Conclusion

Our results indicated that the obesity prevalence in China was still problematic very recently and that we should do more to improve the situation. More public health work is also needed to improve the current status of obesity in school-aged students.
